# Portuguese research on physical education and sport didactics—a critical discussion

**DOI:** 10.3389/fspor.2023.1172815

**Published:** 2023-08-04

**Authors:** Marcos Onofre, João Costa, João Martins, Ana Quitério, Cláudio Farias, Isabel Mesquita

**Affiliations:** ^1^ Centro de Estudos de Educação, Faculdade de Motricidade Humana, Universidade de Lisboa e Unidade de Investigação e Desenvolvimento em Educação e Formação, Instituto de Educação, Universidade de Lisboa, Lisbon, Portugal; ^2^ School of Education, University College Cork, Cork, Ireland; ^3^ Faculdade de Desporto, Universidade do Porto, Porto, Portugal; ^4^ Centro de Investigação, Formação, Inovação e Intervenção em Desporto, FADEUP, Porto, Portugal

**Keywords:** teaching physical education, physical education teacher education (PETE), primary education, secondary education, educational settings

## Abstract

This article presents a discussion of research in Physical Education and Sport Didactics in Portugal. It starts by situating it from an historical perspective, placing the 1980s as the beginning era, mainly based on the studies provided by the two first Physical Education higher education institutes of the country. The initial research, first based on master and doctoral dissertations, progressed to ongoing projects that have been disseminated in international and national journals and books. This development is also reported from the theoretical, conceptual, and methodological perspectives, showing how it has informed the quality of Physical Education and teacher education as the two main research strands to be described, however, acknowledging that a strand on sports coaching and coach education exists. On teaching Physical Education, the article discusses the elements relative to the teacher and to the student, focusing from the immediate and short-term to the distant and long-term events that lead into young adulthood's active lifestyles. In this analysis, research on curriculum and assessment are also reported. On physical education teacher education, the article shows the prevalence of the post-primary Physical Education to argue for the need for more research on primary-level education, and discusses the diverse foci from initial teacher education to in-service education practises. In line with current trends in research, we suggest a set of four features for the future research agenda: (1) addressing short to long-term outcomes of Physical Education; (2) adopting multifactorial and multi-layered perspectives of analysis; (3) embracing inter- and multidisciplinary designs; and (4) taking comparative perspectives within and between European countries, and between Europe and other continents. We conclude that these features need to focus on four levels of integration and cooperation: (a) integration between the research initiatives and the needs of the professional field; (b) integration between research on teaching and learning, teacher education and the curriculum; (c) cooperation between the different national higher education and professional institutions; and (d) integration in the international research agenda by leading and participating in project partnerships which are needed to fully and effectively implement such agenda.

## 1. Introduction

Research in Portugal on Physical Education (PE) and Sports Didactics, since its inception in the 1980s, has experienced an upwards trajectory in terms of scope of covered topics, reach through publications in international peer-reviewed journals, and impact through citations and funding. Yet, there is no review or discussion about it, as non-native English countries are typically under-represented in research reviews [e.g., (1–3)]. This paper contributes to this gap by providing a critical discussion of research on Physical Education and Sport Didactics in Portugal. In doing so, the paper aims to enhance the visibility of and engagement with Portuguese research in the field, with a focus on the formal educational settings (i.e., Physical Education and Physical Education Teacher Education). This is achieved by primarily building on a historical perspective to critically discuss the research on conceptual, paradigmatic, methodological, and empirical perspectives, as essential to move the field forward (4, 5). With this in mind, the paper did not develop from a methodological approach to the literature review. Instead, samples of research are provided to illustrate the PE and Sport Didactics strands that are discussed here. This will also show how the international relevance and recognition of Portuguese research has grown from an inward dissemination process through master and doctoral works to an outward dissemination orientation, favouring even more publications in international peer-reviewed journals and international collaborations. The paper culminates with a proposal of research recommendations aligned to contemporary trends in the field.

## 2. Historical development of sports didactics

In Portugal, research on Sport Didactics significantly began in the mid-1980s and it has concurrently evolved and developed in the sports coaching the educational settings. As the paper intends to primarily focus on the educational setting where all children and youth go through, it can be summarised that research on Sport Didactics has been essentially developed in two areas of study: (1) research on the teaching of physical education (PE); and (2) research on physical education teacher education (PETE). This research has been mainly implemented by the Faculty of Human Kinetics (FMHUL) from the University of Lisbon and by the Faculty of Sport (FADEUP) from the Oporto University. From the beginning, two research trends, one based on the western Europe and EUA tradition (research on Teaching Physical Education) and the other from the eastern Europe tradition (namely, from East German—Sport Didactics research), progressively converged in a quite common agenda.

In Lisbon, as the birth cradle of Sport Didactics research in Portugal, originally in the Faculty of Human Kinetics (FMH in the Portuguese acronym) in the Technical University of Lisbon, most was developed under the Masters Graduate in Education Sciences in the specialisation of Methodology of Teaching in Physical Education. Professor Francisco Carreiro da Costa (6), led by Piéron (6) who integrated the FMH faculty of the master's degree and directed several master theses, and developed a “research school” that successively used the paradigms of process-product, mediation processes, and teacher thinking as conceptual frameworks for the study of physical education and sports education. This research mainly used deductive and quantitative methodologies, where observations of the teacher and student classroom behaviour were preferential indicators. In the 1990s, this type of research was deepened, especially focusing on students (8, 9) and teacher's thinking and knowledge (10, 11), relating to the quality of teaching by combining deductive/quantitative methodologies with inductive/qualitative methodologies. Intensive methods of inquiry have become part of the instruments used in research and became tradition in the next generation of doctoral research (e.g., 12, 13).

In Oporto, this trend was followed in around the same time, starting with process-product, mainly on feedback, and then moving to student-centred research (14). Concurrently, the first 15 years of the 21st century have been marked by the development of studies within the ecological paradigm [e.g., (11, 15)] and interpretivist approaches in both universities. However, previous research traditions are also still visible in some instances, considering the specific epistemological aspects they carry and always dependent on the research questions in evidence.

The accumulated experience allows FMH, currently in the University of Lisbon, to be recognised as an important contributor to the international research on Physical Education and PETE quality, more recently by leading a challenging and ambitious European-based project that is the development of the European Physical Education Observatory (EuPEO) funded by the European Commission (16).

**Figure 1:**
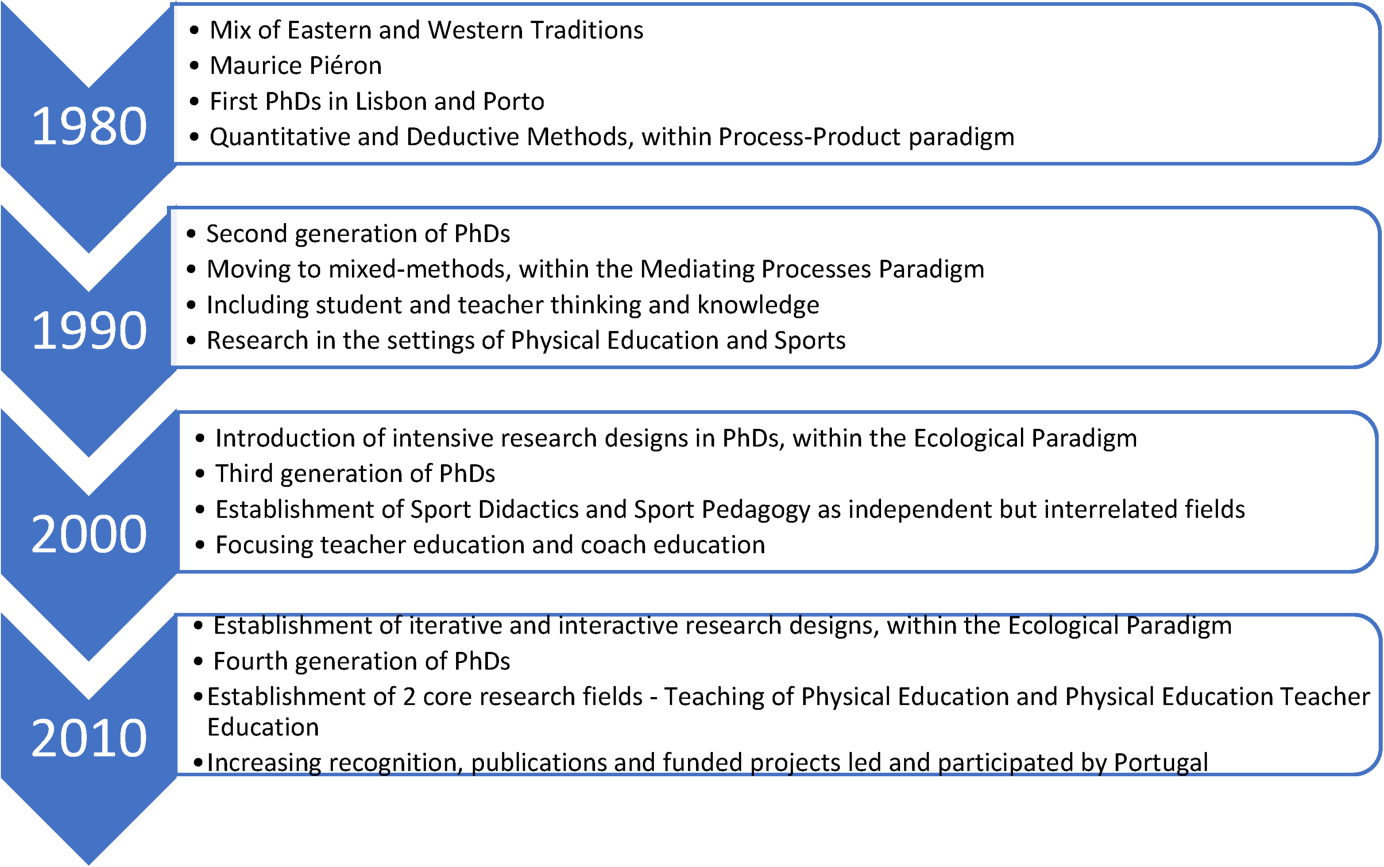
Timeline of research in Sport Didactics in Portugal.

Prominent trends and concepts in Sport Pedagogy hold a strong and critical contribute from the Oporto team, led by Professor Olímpio Bento who got his PhD (1986) at the German Democratic Republic and wrote important documentation on pedagogy and physical education planning (17). Within this group, research on Sport Pedagogy strongly developed with the study of Graça (18) on teachers’ pedagogical content knowledge, with the work of Mesquita (19) on didactics, namely, based on models and teaching strategies in Physical Education and Sport, and with the work of Batista (20) on PETE. In Oporto, the terminology of Sport Pedagogy has been dominant, although there is consideration of aspects traditionally more related to Sport Didactics, namely, concerning the development of the content in learning tasks, lesson plans, and units of learning (Figure 1).

During the last decade, many examples of institutional cooperation between these two schools have been developed. Other research has been developed by other schools as Coimbra University, or Trás-os-Montes and Alto-Douro University.

## 3. Prominent trends and concepts in sports didactics

In Portugal, there is a clear separation between the notions of Sport Didactics and Sport Pedagogy, despite both areas sharing essential authors, theoretical and conceptual frameworks, and research methodologies. In essence, Sport Didactics is considered subject-specific with common aspects typically referred to the operational aspects of designing learning tasks and the lesson plan, and breaking down the learning objectives into assessment and learning tasks, with the respective teaching strategies. Sport Pedagogy is conceived from a broader perspective on the justification of decisions by the teacher on planning, teaching, and assessment that facilitate positive relationships between the students, between the students and the teacher, and between the students and the content, heavily supported by learning theories and embedding other scientific fields (e.g., Sport Psychology) as support knowledge. This is evident not only in how the different teacher education programmes in Portugal offer modules both in Sport Didactics (relative to each subject-content, for instance Basketball or Dance) and Sport Pedagogy but also in the National Coach Education Plan (21) where a unit of learning is titled “Sport Pedagogy and Didactics” framing sports coaching as a student-centred educational process (22).

Notably, research and practise on didactics tends to use slightly different terminology from that in pedagogy, reflecting very specific foci, namely, “didactical organisation of the content,” which would be equivalent to “pedagogical content knowledge.” Furthermore, aligned to the Anglo-Saxonic view, there is also a move from “instructional models” terminology to “pedagogical models” or “model-based practise” (23) to represent, for example, the models of sport education or teaching games for understanding.

As mentioned in the previous section, these two notions are clearly converging on two main research strands where one focuses on the teaching of Physical Education and the other on PETE. These strands are currently more useful in understanding and addressing Portuguese research rather than feeding the discussion on whether it is aligned to Sport Didactics or Sport Pedagogy. It is also important to reaffirm that there exists another set of Sport Didactics research, more focused on the sports coaching field, covering a wide range of topics that mostly fall under the scope of the coaching environment, coach methodology, and coach education. However, this article prioritises the educational settings, and the authors suggest that another similar paper can be delivered to focus the research on the sports coaching setting.

## 4. Examples of application in educational contexts

Following the previous section, we will refer to the application of Sport Didactics research in the educational context from the perspective of school-teaching and that of teacher education.

### 4.1. Teaching physical education

Sports Didactics research on the teaching of Physical Education started from a process-product perspective, moving to a more recent one informed by the ecological paradigm (11, 15, 24), largely influenced by the Anglo-Saxonic research pushing this agenda (25). As such, this strand of research has informed current knowledge on different classroom dimensions, namely, those more related to the teacher or to the student. This research tends to focus on the more immediate setting of the classroom and the lesson, or at less immediate settings as the social and professional environments of teachers and, occasionally, on more long-term perspectives on the influence of Physical Education in the youth lifestyles. Samples of this overview are discussed in the following paragraphs.

Regarding the teacher, initial studies have refuted the idea of teaching as an idiosyncratic or universal model (26) to establish the essential dimension of the teaching behaviour and teaching methods to support diverse domains of student learning (26, 27). This line of research has embedded the notions of instructional and curricular models to expand the understanding of the teachers’ didactical and pedagogical behaviours in scaffolding students learning. In this regard, Farias (28, 29) ran the first Portuguese study in Sport Education Model (SEM) that provides a year-long, in-depth examination of the scaffolding processes over four consecutive SEM seasons to find that the scaffolding of the student–coaches’ instructional leadership was a non-linear process dependent on teacher-controlled contingencies. Also on SEM, it has been found that different levels of teacher guidance and learners' instructional responsibility are necessary when teaching tactic (30) as well as positive student perceptions on the educational value from the Physical Education teacher (31).

However, it was established that looking only at the teacher behaviour is highly aligned with a behaviourist and narrow view that compromises a more comprehensive understanding of the teaching and learning process (25). This recognition pushed the relevance of advancing to the mediating factors paradigm as an explanatory model of the interaction mediators between teaching and learning. From there, as pointed by Onofre (24), the features of student and context started to be considered from the ecological perspective, looking into the classroom as a habitat where academic, organisational, and social agendas emerge and mutually interact with each other. Such research, as in related international research [e.g., (32)], shows how the teacher manages the classroom ecology for the students’ engagement, concluding that the better the teachers work in integrating the students’ agenda in the program of action, the more the students engage with the learning tasks and the better are their perceptions. To achieve such level of integration, Costa et al. (33) demonstrated how the collective and collaborative work of the Physical Education department supported and translated into better integration strategies by the teacher as observed in teaching practise and perceived by the teachers.

This line of research on the teachers' perspective has also looked at the assessment practises in the classroom, considering both the broader environment and the relation with the curriculum (34). According to Quitério et al. (35), from a conceptual review on the historical and current trends for PE assessment, since mid-1980s, the role of assessment within Physical Education in Portugal was mainly focused on measuring the learning goals, based on quantitative methodologies. The discussions related to the purpose and need of a systematic Physical Education assessment in Portugal have gained particular attention during the 1990s (36). Since then, much like what was being argued for internationally at around the same time (37), Portugal adopted a perspective of authentic assessment (38) and a strong focus on formative assessment as a fundamental strategy to enhance student learning (35). In line with this curriculum expectation, some studies show that teachers are aware of the important and crucial role of formative assessment (39, 40). Yet, the way teaching is organised among Portuguese schools and the overall educational system place internal and external constraints on the quantity and quality of their formative assessment practises, as there are different perspectives and conceptions among teachers (40). Considering this misalignment, Costa (15) concluded on the fundamental importance of the Physical Education department's collaborative work to integrate PE assessment practises, which are systemically aligned across curriculum, assessment, and didactics and pedagogy as essential to ensure quality Physical Education experiences to all children and youth.

With regards to the students, the majority of research tends to focus on their thoughts, attitudes, and learning, which is strongly advocated (2, 3, 41), and is particularly salient in current research on social justice movements (42), which is slowly emerging in Portuguese research. Most research shows a positive image of Physical Education from students (9, 43). Yet, their perception about the aims of Physical Education varies. Pereira (9) verified that having fun and recreation were the most privileged aims for students, where other studies reported health and fitness (8, 43) and some others found that students privilege practising sports under a learning-oriented perspective (44). When the focus is on the self-perceptions of students, it has been established that those with a more positive view also understand better and engage more with learning (43). Notably, enjoying and valuing PE seems to be associated with having higher perceived competence (45, 46).

When focusing on student learning, research has also addressed how they experience their instructional environment from the perspective of motivation and of the instructional models. On motivation, results show higher levels of mastery orientation compared to ego orientation (47). Martins (45) found that active adolescents were characterised by having a high mastery orientation and a low performance-avoidance orientation, and perceived their PE classes to be mastery-involving, as opposed to inactive adolescents. When looking at the research on instructional models, it has been shown that a focus in pupils’ autonomy, as in the SEM, has different effects compared to teacher-centred models as the Direct Instruction Model (48). SEM has shown a strong impact for all pupils in sport performance on volleyball (49) and track and field (50). Considering the students' perception about the development of personal and social responsibility in both Sport Education and Direct Instruction, SEM shows a greater influence on the students’ personal and social responsibility (51). The research on the instructional models provides evidence that integrating (mixing) models has an advantage for pupils’ learning, for example, between the Sport Education and the Invasion Games Competence Model, especially for girls and low skill–level students (52). The same is true for the pupils’ improvement in game performance and understanding (53). The examination of the impact of a hybrid combination of SEM and the Step-Game-Approach on students’ gameplay performance in volleyball, considering their gender and skill level, showed an improvement both for boys and girls on their performance and that the lower skilled students achieved greater gains than those of higher skill during the unit (54). Still on the integration of models, SEM and Step-by-Step Game have been the most examined ones in Portugal. Mesquita et al. (14) found that girls and low skill–level students seemed to take more benefit from the Step-by-Step Game Model alone, than boys or high skill–level students. It has to be noted that the critical contribution of this line of research is addressing critical questions missing in previous SEM literature where Farias et al. (28, 29) examined and intervened on student behaviours to promote a democratic, inclusive, and participatory focus. The authors found a close interrelatedness between game competence development, trajectories of participation, and sense of membership, with the restructuring of power relations and the sharing of knowledge and investment of dominant and higher-skilled students towards more inclusive team goals.

Despite the focus on the more short-term learning achievement of students, Portuguese research has also sought to understand its development in longer timeframes as a methodological approach typically scarce in PE research on student learning (1, 55). Farias et al. (56) examined game performance according to the tactical structures of invasion games throughout three consecutive model-based units. Farias et al. (28, 29) during a year-long study also observed that as the student–coaches developed knowledge of content and instruction, they became increasingly self-assisted in the conduct of the learning activities. Looking at the long-term influence of Physical Education in physical activity (PA) participation, the PA levels of adolescents are low, decline with age, and are worst in girls compared to boys as they are consistently low and stable over the years (2000, 2006, 2010, and 2014) (57). Several studies in Portugal have focused on understanding the PA correlates and how these factors might be taken into account for promoting active lifestyles (47, 58–60). At the individual and psychological level, in addition to PE attitude, PA attitude, and goal orientation, it has been shown that intrinsic motivation, perceived competence, and self-efficacy are all important correlates of overall PA levels of children and adolescents (45). Moreover, active adolescents tended to have better academic achievement (45, 47, 61) and more stable support from family (45) and friends (45, 62).

Furthermore, Farias et al. (63) has demonstrated the concurrent evolution between game performance and patterns of game involvement over extended time, i.e., three consecutive sport teaching units. This longitudinal approach has made possible to capture the global evolution of the students’ ability to actively participate in the game, implicitly containing evidence of equity of participation in the learning experiences. Research on the influences of structural and contextual elements of the teaching–learning process on opportunities for students to participate in sport-based activities was also studied (64). It was possible to perceive variances in the opportunities for participation given in PE classes to different groups of students (higher or lower skilled boys and girls), revealing the most favourable contexts of practise (competition or team practise events) to be privileged by teachers.

In connection with the development of physical literacy and students’ willingness to participate in physical activity throughout life conducted in OPorto, the study by Farias et al. (65) was the first research to carry out a 4-year follow-up study to ascertain the transformative potential of a model-based PE curriculum. The project developed an embodied self-regulated perception on the need to proactively nurture in the students, in PE classes and outside the school context, social justice, equity, and inclusion contexts. There was also evidence of the transfer of these social skills into inclusive proactive citizenship attitudes, in the context of youth sports coaching (greater tolerance for their peers’ performance errors), and other areas of life (dynamisation of local clubs of social solidarity and entrepreneurship).

### 4.2. Physical education teacher education (PETE)

The impact of the above discussed research is visible in today’s PETE programmes that place considerable relevance in the acquisition and performance of classroom management behaviours sustained in procedures relative to instruction, organisation, discipline, and climate, aligned to instructional and curricular models or informed by teaching styles. Moreover, this preparation is mostly focused on the dimension of physical activities and health-related fitness, leaving less addressed the dimensions of knowledge and values from a didactical and pedagogical perspective on how to plan, teach, and assess. Essentially, as Calderón and MacPhail (66) suggest, there is a great opportunity for PETE programmes (in Portugal) to blend different paradigms and models of teacher education beyond the mainstream behaviourist technocratic approaches towards critical models as well. In doing so, the breadth of research in Portugal on PE and Sport Didactics may continue to contribute to the development of PETE in blending models through a range of signature pedagogies (67).

Most of the research on PETE focuses on the post-primary level. Neves (68) completed the first PhD in Portugal, University of Aveiro, on primary education teachers’ knowledge and perception of the PE curriculum as part of professional development. According to Neves (69), the preparation of Portuguese professionals to deliver Physical Education at the primary level is incredibly scarce, which is a research area mostly led by University of Coimbra. As for the initial preparation for primary-level PE, research has addressed the student–teachers’ perceived value on training strategies to teach Physical Education (70). At the primary school in-service level, research has explored project-based CPD models with extending periods of time during the school year and alternating school-based and out of school integrated practises (71), as well as their knowledge and self-efficacy beliefs (72).

Likely, because in Portugal the Physical Education teachers at the post-primary level are trained as specialists, research on post-primary PETE is more prominent in volume and scope. Clearly, a fundamental aspect for Portuguese research on PETE is the school placement period, which has captured supervisory practises during this fundamental period (73–75) and its influence as a professional development source (76). These studies have confirmed the determinant value of school placement and types of supervision practises engaged by the tutors for the student–teachers’ professional development and teaching behaviours. Furthermore, the supervisors’ role as influenced by the context (77) or their identity construction (78) and levels of cooperation (76, 79) represent another research focus for the school placement. This line of research shows the challenges that supervisors face in undertaking supervision as an individual and collaborative role, which is shaped by political and institutional (from schools and universities) constraints. However, research on PETE school placement also looks at the student–teachers considering their beliefs (80–84), their identity formation (85), and their learning process (86). Overall, this line of research shows progression and deepening of knowledge of the student–teachers throughout placement and the narrowing of the theory–practise gap as positive outcomes of their school placement period. It is also noteworthy to highlight that the professional development of newly qualified teachers has been a focus of research in Porto (87).

From a practical standpoint, such research in PETE has translated into clearly identifiable practises. For example, in Oporto, the practical content is delivered in a combined approach through “pedagogical models” (e.g., SEM) and the “pedagogical content knowledge” (e.g., step-game-approach) (88). Furthermore, longitudinal research has been carried out to uncover the professional development of student–teachers. This has revealed not only the pedagogical challenges experienced by student–teachers during the application of the model-based PE curriculum in school placement (89) but also the alignment between the training received in the first-year course and the pedagogies effectively enacted in school placement from the point of view of view of social learning theories (90). Recently, Farias et al. (91) studied the “Multi-system influences on Physical Education preservice teachers’ teaching practise in pandemic times” to uncover a complex web of macro-, meso-, exo-, micro-, and individual system elements that impact on the teaching practises of student–teachers. There, the need for PETE programs was stressed to endow student–teachers with flexible pedagogical skills and to flexibly and conjointly apply different student-centred pedagogies through explicit training on technological pedagogical content knowledge and digital skills.

Following international research on the post-primary in-service stage, and as requested by Cochran-Smith and Villegas (92) to develop a chain of evidence between teacher education and student learning, it is rare to find Portuguese research that seeks to link the Physical Education teachers’ professional development with their pedagogical practises (13, 15, 87). More regularly, research on in-service teachers addresses teachers’ engagement with learning communities (32, 93) or their perception, value, and needs of CPD provision (94). At a broader educational perspective, along with the research and professional practises in PETE (95), Portuguese research seems to have embraced the notion of learning communities and communities of practise although it is not clear how much schools and teachers are aware and seeking opportunities to implement them in their everyday practise.

## 5. Developing and improving future research

Currently, as in the international context, Portugal has been embracing the concept of Physical Literacy (96), mostly for the educational context. While this is starting to be a discussed concept as intrinsically aligned to the Portuguese PE curriculum aims (97), research is slowly taking it in the mainstream discourse with some beginning doctoral work and published research from Lisbon (98). While the present Physical Education curriculum and assessment already considers what are deemed inherent elements of physical literacy, there is the need to align the previous and future research according to the physical literacy conceptual dimensions. Indeed, Portugal has contributed to this conceptual discussion with some of the existing reviews (2, 3). Given the prominence of physical literacy in the current literature, its conceptual discussion needs to be considered by future research not only for the context of the classroom but also looking ahead from a perspective of lifelong participation in physical activity as a desirable outcome of Physical Education. According to Onofre (97), this aligns with a notion of “active life,” which is nowadays reflected in the image of an “active citizen” as someone who takes ownership for the improvement of society and the environment, adopting an active and healthy lifestyle and who supports the generalisation of these behaviours to the fellow counterparts.

Such a view, the historical developments of research on Physical Education and Sport Didactics (with a focus on educational settings), and the alignment with international research as demonstrated throughout the paper, in our perspective, pushes the future Portuguese research agenda at an international level to adopt at least four fundamental features of

(1) Addressing short- to long-term outcomes of Physical Education,
(2) Adopting multifactorial and multi-layered perspectives of analysis,
(3) Embracing inter and multidisciplinary designs, and
(4) Taking comparative perspectives within and between European countries, and between Europe and other continents.

Looking at the first feature, we believe that, based on the reported literature, Portuguese research has been sharing the attention between the immediate events and outcomes that occur at the time of the lesson and those that are observable at later stages in life as related (positively or negatively) with past educational experiences. However, this notion also needs to be critically implemented for the full education continuum where Primary Education research in Physical Education is fundamentally lagging both for teaching and teacher education. This aspect relates to the second feature of adopting multifactorial and multi-layered perspectives of analysis, while pushing the boundaries forward.

Boundaries can be pushed towards asking if the non-physical/non-psychomotor outcomes typically attributed as specific to PE (e.g., resilience, teamwork, fair-play, respect) are also being effectively achieved during the lesson and in later stages in life from a perspective of physical literacy. However, to adopt such multifactorial and multi-layered perspectives in Physical Education and Sport Didactics research also means to fully embrace the ecological paradigm (and other contemporary paradigms as post-structuralism or critical research) and look at other settings and variables that are not exclusive to the classroom, but which have an interactive potential to foster or hinder the classroom educational experiences from the formal curriculum. It has to be noted that, in our view, current “publishing metrics” and academic career outcomes tend to facilitate univariate, short-term, and simplistic analysis to favour more publication outputs. However, this is against the best interest of a broad and effective understanding of personal features, contextual variables, procedural behaviours, and time scales that affect the development of physical literacy as a multidimensional concept in itself. In addition, the use of methodological eclecticism brought along the use of sophisticated research designs of a longitudinal nature, allowing the understanding of the pedagogical phenomenon throughout the process. This is particularly important in meeting the epistemological nuances of most teacher education programs, involving pedagogical processes that move beyond the view of learning teaching methods in the university to their practical application in the school placement. Additionally, the use of mixed and more varied research designs, such as ethnography and action-research with transformative purposes, makes possible not only to understand in-depth the research problems as pedagogically, historically, and culturally situated but also to intervene directly in improving the quality of teachers’ (and future teachers’) practises.

As for the third feature of embracing inter- and multidisciplinary designs, it represents the medium through which the previous features can be better developed by combining different expertise and theoretical frameworks in shared research projects. Most of this type of research seems to come from the interplay between Sport Psychology on motivation and Sport Didactics on teaching and learning variables and constructs. However, the multidimensional facet of teaching and learning, and of physical literacy as a driving concept, requires more fields and methodologies to connect under combined research designs.

Lastly, there are very few studies that compare the Portuguese reality with any other, or that compare sets of European countries with sets of countries from other continents. In Portugal, since the early 1990s, the Physical Education curriculum (99–102) has been consistent and refined in adding sets of norms, guidelines, and standards. The regular refinement clarifies the concept and general aims of Physical Education, implicitly towards this notion of physical literacy, through a clear, consistent, and coherent progression from early years’ education to the end of secondary education. The PE aims are based on three core values of multidimensionality, eclecticism, and inclusiveness. This set of documents needs to be compared to other European Physical Education curricula in all structural dimensions and in its implementation. The EuPEO (16) offers one example that takes all these four features, and it is hoped that its structure and outcomes help shape and inform this research agenda.

## 6. Concluding remarks

As a synthesis, research in Portugal on Physical Education and Sport Didactics, with a focus on educational settings, was deeply developed during and after the 1980s, mainly based on the studies provided by the two main Faculties of the country, FMHUL and FADEUP, initially with an inwards dissemination perspective based on master and doctoral dissertations. This has clearly evolved and matured to an outwards dissemination and collaboration approach resulting in ongoing collaborative projects, which have been disseminated in international and national peer-reviewed journals, conferences, and books. At the beginning, research focused on teaching, following the process-product paradigm, then moving to mediational and ecological paradigms, and more recently showing signs of adopting critical and social justice approaches. Research also moved to address initial teacher education and continuing professional development, namely, based on the social-constructivist perspective, having clearly adopted current views of learning communities that are still to be shared with the practitioners through critical approaches. That initial research allowed us to collect significant evidence about the quality of Physical Education and teacher education that is now being used to inform practises.

Later, considerable research on curricular development was established, with a strong boost from the curricular implementation of student-centred pedagogical models based in Oporto, expressing a marked concern for the dimensions of inclusion and provision of equal participation opportunities to PE students independently of their background individual characteristics. The wide scope of this research has allowed advancing knowledge simultaneously on optimising pedagogical strategies to improve teaching and for creating a more coherent and significant connection between such (student-centred) curriculum and student development within a multidimensional educational perspective (motor, social emotional, and cognitive), under the Physical Literacy agenda, which has naturally been adopted by research in Portugal and will continue so.

As we provide a set of four features to shape the future Portuguese research agenda, we hope it becomes more cohesive in four types of integration and cooperation: (a) integration between the research initiatives and the needs of the professional field; (b) integration between research on teaching and learning, teacher education, and the curriculum; (c) cooperation between the different national higher education and professional institutions, especially schools; and (d) integration in the international research agenda by leading and participating in project and community partnerships.
